# Correlates of Physical Activity Among Older Adults of Low Versus High Socio-Economic Status

**DOI:** 10.1093/geroni/igaf122.660

**Published:** 2025-12-31

**Authors:** Olivia Malkowski, Jessica Harvey, Nick Townsend, Mark Kelson, Charlie Foster, Max Western

**Affiliations:** Yale University, New Haven, Connecticut, United States; University of Bath, Bath, England, United Kingdom; University of Bristol, Bristol, England, United Kingdom; University of Exeter, Exeter, England, United Kingdom; University of Bristol, Bristol, England, United Kingdom; University of Bath, Bath, England, United Kingdom

## Abstract

Understanding socio-economic differences in the factors influencing physical activity among older adults is essential for developing comprehensive interventions. We aimed to quantify the associations of modifiable correlates and determinants with physical activity among UK-based older adults of low versus high socio-economic status (SES). In this systematic review and meta-analysis, we searched MEDLINE, Embase, Web of Science, CENTRAL, and Scopus from inception to December 2023, for peer-reviewed studies published in English, investigating associations between a modifiable factor as an independent variable and physical activity as a dependent variable, by SES, in community-dwelling UK older adults aged 60+ years. Random effects meta-analyses were performed separately for people of low and high SES. Risk of bias was assessed with the Mixed Methods Appraisal Tool. This study was registered with PROSPERO (CRD42022351708). Searches identified 11,472 references; seventy-seven studies met the selection criteria, of which fifty-one contributed to meta-analyses (N range = 134–29,280). Of the exposures positively associated with physical activity, physical function, social participation, and perceived general health had the largest effect sizes (standardised mean difference [SMD] range = 0.53–0.81; I-squared statistic range = 54.81–91.00%). Estimates were comparable among low- and high-SES older adults, except for built physical activity facilities, walking and cycling infrastructure, and less smoking, which were positively associated with physical activity only among low-SES individuals. Our results suggest researchers need to better understand discrepancies in the prevalence of the assessed correlates (e.g., fewer low-SES participants reported good physical function) to inform policies that reduce inequalities in older adults’ physical activity levels.

